# Sex Differences in Neuromuscular Adaptations Following 12 Weeks of Kettlebell Swing Training

**DOI:** 10.7759/cureus.70551

**Published:** 2024-09-30

**Authors:** Evaldo Rui T Santos Junior, Belmiro F De Salles, Ingrid Dias, Roberto Simão, Jeffrey M Willardson

**Affiliations:** 1 Gymnastics, Federal University of Rio de Janeiro, Rio de Janeiro, BRA; 2 Health and Human Performance, Montana State University Billings, Billings, USA

**Keywords:** crossfit, kettlebell swing, physical fitness training, resistance training, sex differences

## Abstract

The purpose of this study was to investigate whether there are sex differences in strength, power, and muscular endurance gains following a 12-week kettlebell swing protocol. Twenty-eight men (29.4 ± 7.3 years; 176.4 ± 6.2 cm; 80.1 ± 11.0 kg; 146.0 ± 22.3 kg in deadlift; 1.8 ± 0.2 kg/body mass deadlift relative strength) and 25 women (32.3 ± 5.5 years; 162.5 ± 7.5 cm; 66.2 ± 9.7 kg; 92.4 ± 17.3 kg in deadlift; 1.4 ± 0.2 kg/body mass deadlift relative strength) participated in this study. Measures of strength in the deadlift, power in the countermovement jump, muscular endurance in the deadlift, and workout of the day (WOD) were obtained before and following six and 12-week time points. Both sexes showed pre- to post-test differences in strength (p < 0.001; one repetition maximum (1RM) improvement of 12.6% for men and 11.7% for women), power (p < 0.001; jump height improvement of 12.9% for men and 6.8% for women), and muscular endurance deadlift test (p < 0.001; improvement in deadlift repetitions of 24.5% for men and 29.2 for women), and only men improved performance in DT WOD (p < 0.001; WOD improvement of 21.3% for men and 1.3% for women). Furthermore, men had greater gains in the power test (p = 0.02) and in the DT WOD endurance test (p = 0.04). Kettlebell training appears to be an effective strategy for increasing strength, power, and endurance in both men and women, but considering the lower responses in power and DT WOD, other strategies should also be considered to optimize women's results in these tests.

## Introduction

Differences in performance between sexes have been extensively investigated in the scientific literature. Usually, men are stronger and more powerful than women, whereas women tend to resist fatigue more than men both in isometric and dynamic actions [1,2]. The origin of these differences can be explained from different points of view, such as the amount of muscle mass, muscle morphology, skeletal muscle gene, hormonal differences, substrate use, and neuromuscular activation, among others [1-3].

The explanation for the difference in strength and power between sexes is generally attributed to the greater amount of muscle mass and larger muscle fibers in men versus women [1,4,5]. The higher concentration of muscle mass in the lower limbs versus the upper limbs in women explains why the differences for the lower body are smaller versus those for the upper body between sexes [4]. In addition, men have greater distribution percentages for Type II, MHC II, IIA, and IIX fibers, greater area percentages for Type II, IIA, MHC IIA, and IIX fibers, and greater Type II/I and Type IIA/I fiber area ratios [5].

The main mechanisms responsible for the difference in fatigue resistance between men and women are normally attributed to muscle mass, substrate utilization, and muscle morphology [1-3]. The first is related to greater muscle mass in men than in women, which leads women to use less absolute muscle strength when the same relative work is performed [3]. Furthermore, greater muscle mass and strength in men involves a higher muscle oxygen demand and play an important role in limiting perfusion and oxygen supply [1, 3]. Regarding substrate utilization, men appear to have a greater glycolytic capacity than women, due to the greater activities of common glycolytic enzymes such as pyruvate kinase, glycogen phosphorylase, phosphofructokinase (PFK), and lactate dehydrogenase (LDH), suggesting a greater potential for beta-oxidation of fatty acids for metabolism in females [3]. These differences may be related to the third explanation (muscle morphology), considering that men have larger type II fibers than women, which leads women to have greater Type I and MHC I distribution percentages, greater Type I and MHC I area percentages, and greater Type I/II fiber area ratios, which may explain the sex differences in fatigability [5].

Another aspect that has been the subject of study is whether sex differences impact neuromuscular adaptations following a period of resistance training. In a systematic review, the authors found that men and women adapt to resistance training in a similar way for lower-body strength, but, possibly due to a lower initial level of fitness, women appear to have a higher capacity to increase upper-body strength than men [6]. For power gains, some results suggest there is no difference between sexes, while others show superior adaptations for men depending on the context. For example, in one study men and women increased upper-body power similarly, but men responded with greater absolute gains than women for lower-body at 40 and 60% of one repetition maximum (1RM) [7]. Another study showed similar power improvements between men and women for lower-body, but men had greater power gains at 80% of 1RM [8]. For muscle endurance adaptations, it is possible to find results revealing no difference between sexes [9], while other studies suggest that women have greater increases than men [10, 11]. Regardless of the outcomes, studies that investigated sex differences in neuromuscular adaptations focused mainly on traditional resistance training exercises, such as the leg extension, back squat, leg press, bench press, and chest press [7, 9], and the investigation of alternative tools and exercises is lacking.

An alternative resistance training implement that has increased in popularity in recent years is the kettlebell [12], which is a cast iron weight comparable to a cannonball with a handle [13]. This design allows its center of mass to extend beyond the hand, which favors ballistic movements such as the swing exercise [14], considered a fundamental and frequently studied exercise in kettlebell training [13]. Considering that physiological sex differences could potentially influence exercise prescription and the subsequent adaptation experienced [15], a better understanding of how men and women adapt to kettlebell swing training can contribute to best practices in program design with this resistance training tool. In this sense, the purpose of this study was to investigate whether there are sex differences in strength, power, and muscular endurance gains following a 12-week kettlebell swing protocol.

It was hypothesized that there would be no sex differences in strength, power, and muscle endurance gains. This hypothesis was justified by the fact that strength and power tests were performed in lower limb exercises (deadlift and countermovement jump, respectively), and the differences in neuromuscular adaptations seem to be significant when exercises for upper limbs are used, with no differences for lower limbs [6].

## Materials and methods

Participants

Twenty-eight men (29.4 ± 7.3 years; 176.4 ± 6.2 cm; 80.1 ± 11.0 kg; 146.0 ± 22.3 kg in deadlift; 1.8 ± 0.2 kg/body mass deadlift relative strength) and 25 women (32.3 ± 5.5 years; 162.5 ± 7.5 cm; 66.2 ± 9.7 kg; 92.4 ± 17.3 kg in deadlift; 1.4 ± 0.2 kg/body mass deadlift relative strength) participated voluntarily in this study (Table 1). All subjects continued to perform the CrossFit® (CrossFit, Inc., Washington DC) program during the intervention period. To be included, subjects had to have at least six months of experience with kettlebell training and an extreme conditioning program (at least three times a week with one-hour sessions, performing at least four exercises per session, and at least three sets per exercise); they must be between the ages of 18 and 50 years. The subjects were not included in the study if they had answered "yes" to one of the Physical Activity Readiness Questionnaire (PAR-Q) items [16] and had any functional limitations or injuries that would affect the tests or performance during the training sessions. The study was approved by the Ethics Committee of the Clementino Fraga Filho Teaching Hospital of the Federal University of Rio de Janeiro (approval number: 86008418.6.0000.5257) which is an institution affiliated with one of the authors, and all subjects gave their written informed consent to participate in the experiment.

**Table 1:** Baseline characteristics of the participants (mean ± SD) MG = male group; FG = female group; RM: repetition maximum

Groups	Age (years)	Height (cm)	Body mass (kg)	Deadlift 1 RM (kg)	Relative strength	Training experience (years)
MG (n = 28)	29.4 ± 7.3	176.4 ± 6.2	80.1 ± 11.0	146.0 ± 22.3	1.8 ± 0.2	2.3 ± 1.3
FG (n = 25)	32.3 ± 5.5	162.5 ± 7.5	66.2 ± 9.7	92.4 ± 17.3	1.4 ± 0.2	2.5 ± 1.1

Design and procedures

To investigate possible sex differences in strength, power, and muscular endurance gains following a 12-week kettlebell swing protocol, 53 subjects (28 men and 25 women) and CrossFit® practitioners participated in this study. The kettlebell protocol was performed twice a week on non-consecutive days. Measures of strength in the deadlift, power in the countermovement jump, muscular endurance in the deadlift, and workout of the day (WOD) were obtained before and following six- and 12-week time points.

Power Rest

Muscle power was assessed with the countermovement jump (CMJ) test. Subjects performed three sets of 10 repetitions of a general warm-up containing two exercises, the hip hinge exercise with a stick and the ground-to-overhead exercise (men with a 10 kg plate and women with a 5 kg plate). Then, the subjects performed two CMJs with hands placed on the hips with a five-minute rest interval between trials, starting in the standing position and performing extension of the hips, knees, and ankles when they were in the air during a jump. Subjects were instructed not to bend their knees, to try to touch their feet simultaneously on the ground, and to jump as high as possible, and the highest one was recorded. The assessments were performed with the My Jump 2 app (Apple Inc., Cupertino, CA), which shows high reliability (intraclass correlation coefficient (ICC) = 0.997, 95% CI: 0.996-0.998, P < 0.001) and almost perfect correlation (r = 0.995, P < 0.001) when compared with a force platform [17]. To register the CMJ, the researcher lay prone on the ground holding an iPhone 6 (Apple Inc.) recording each jump from a frontal plane, at a distance of 1.5 m. The take-off phase was obtained in the first frame in which both feet were off the ground, and the landing phase was considered the first frame where at least one foot was touching the ground [17]. From these two frame selections, the app calculated the jump height (reported in centimeters) from flight time.

One Repetition Maximum Test 

Maximum strength was assessed with 1RM testing in deadlift exercise. Subjects performed a specific warm-up with progressive loads that consisted of five reps at 50%, four reps at 60%, and three reps at 70% of 1RM. Maximal strength testing was performed approximately 10 minutes following the CMJ test. The subjects were instructed to keep their back straight, keep the bar close to the body throughout the movement, fully extend the hip and knee, and return with the bar without releasing it on the floor. Verbal encouragement was provided during all 1RM attempts, and each subject had up to four trials separated by a three- to five-minute rest period. Progressive increments were made in the load following each successful performance until a failed attempt occurred [18]. The individuals had to finish the concentric phase with hips and knees completely extended [19]. For standardization purposes, subjects had to perform the conventional deadlift style (with the grip they preferred).

Muscle Endurance Test

Muscle endurance was assessed by performing the deadlift using 50% of 1RM for as many repetitions as possible with proper technique with the plates touching the ground before a new repetition began [20]. The same criteria of the 1RM test were used for a repetition to be considered valid. For standardization purposes, subjects were instructed to only use the double overhand grip (palms facing backward). The muscle endurance test was performed approximately 10 minutes following the 1RM test.

Performance in CrossFit® WOD​

Approximately 10 minutes following the muscle endurance test, subjects performed a CrossFit® WOD called DT. The DT WOD consisted of performing five sets of 12 deadlifts, nine hang power cleans, and six push jerks as fast as possible (men using 70 kg and women using 47.5 kg). In the present study, individuals performed a modified version of DT and had to complete as many sets as possible of 12 deadlifts, nine hang power cleans, and six push jerks in 10 minutes (men using 40 kg and women using 25 kg).

Training Protocols

Under a certified kettlebell instructor's supervision, individuals performed 12 sets of 30 seconds doing as many kettlebell swing reps as possible, alternating with 30 seconds of rest. This training protocol has already been used in previous studies [20-22]. The subjects were instructed to keep the back straight; project the hips backward, not downward; focus on powerful hip extension, not arm movement; fully extend the hips and knees, with the body forming a straight line at the end of the movement; at the top of the movement, the kettlebell should be an extension of the arms, which should be straight and relaxed. According to the guidelines recommended by Tsatsouline in Enter the Kettlebell [23], men started with 16 kg and women with 8 kg, and throughout the training period, men reached 24 kg and women 16 kg.

Statistical analysis

A two-way repeated measures ANOVA with Bonferroni post hoc analysis was used to verify the differences in power, maximum strength, muscular endurance, DT WOD adaptations, and sex differences in the test performance. A T-test for independent samples was used to verify potential differences in the percentage change (Δ%) between sexes from pre- to post 12 weeks of training in power, maximum strength, muscular endurance, and DT WOD. The change score divided by the standard deviation of the change score was used to calculate the effect size (ES) [24], and the scale proposed by Rhea for determining the magnitude of ES in strength training research was used [25]. Statistical analyses were performed with IBM SPSS Statistics software, version 26 (IBM Corp., Armonk, NY), and the statistical significance was set at p < 0.05.

## Results

**Figure 1:**
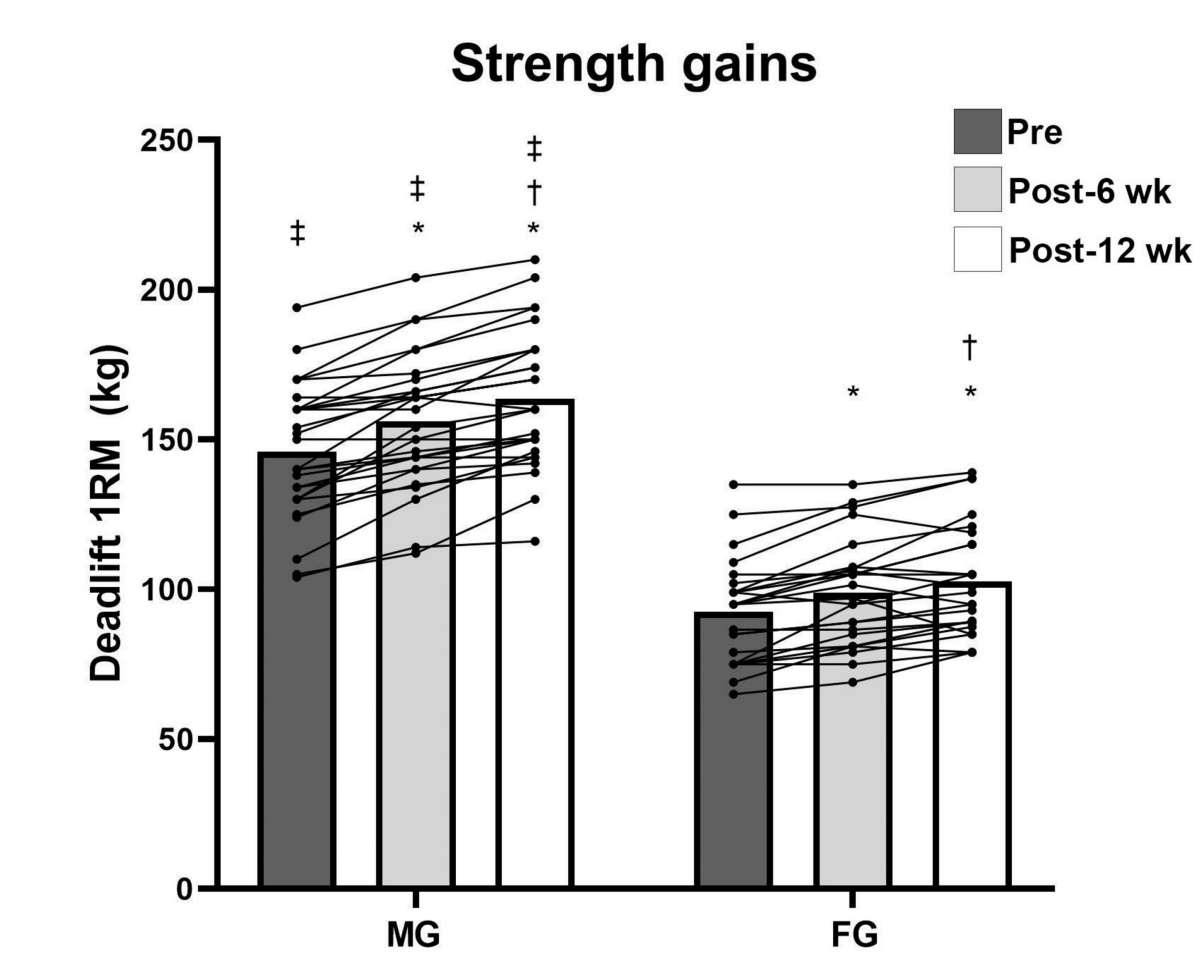
Deadlift one repetition maximum (1RM) pre- and post training for male group (MG) and female group (FG) *Significant difference from pre- to post training; †Significant difference from the sixth to 12th week (wk) of training; ‡ Significant difference between the sexes

**Figure 2:**
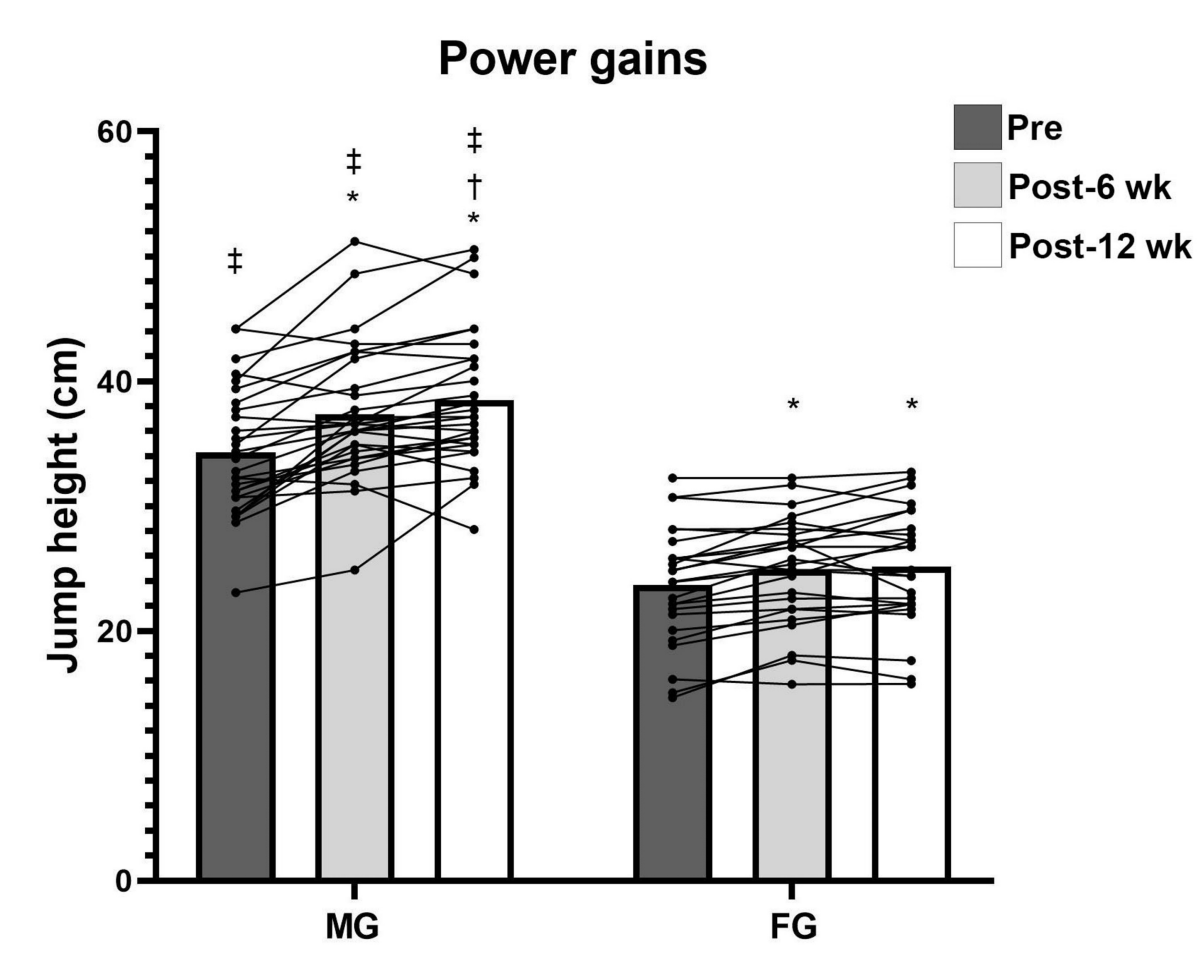
Jump height pre- and post training for male group (MG) and female group (FG) *Significant difference from pre- to post training; †Significant difference from the sixth to 12th week (wk) of training; ‡ Significant difference between the sexes

**Figure 3:**
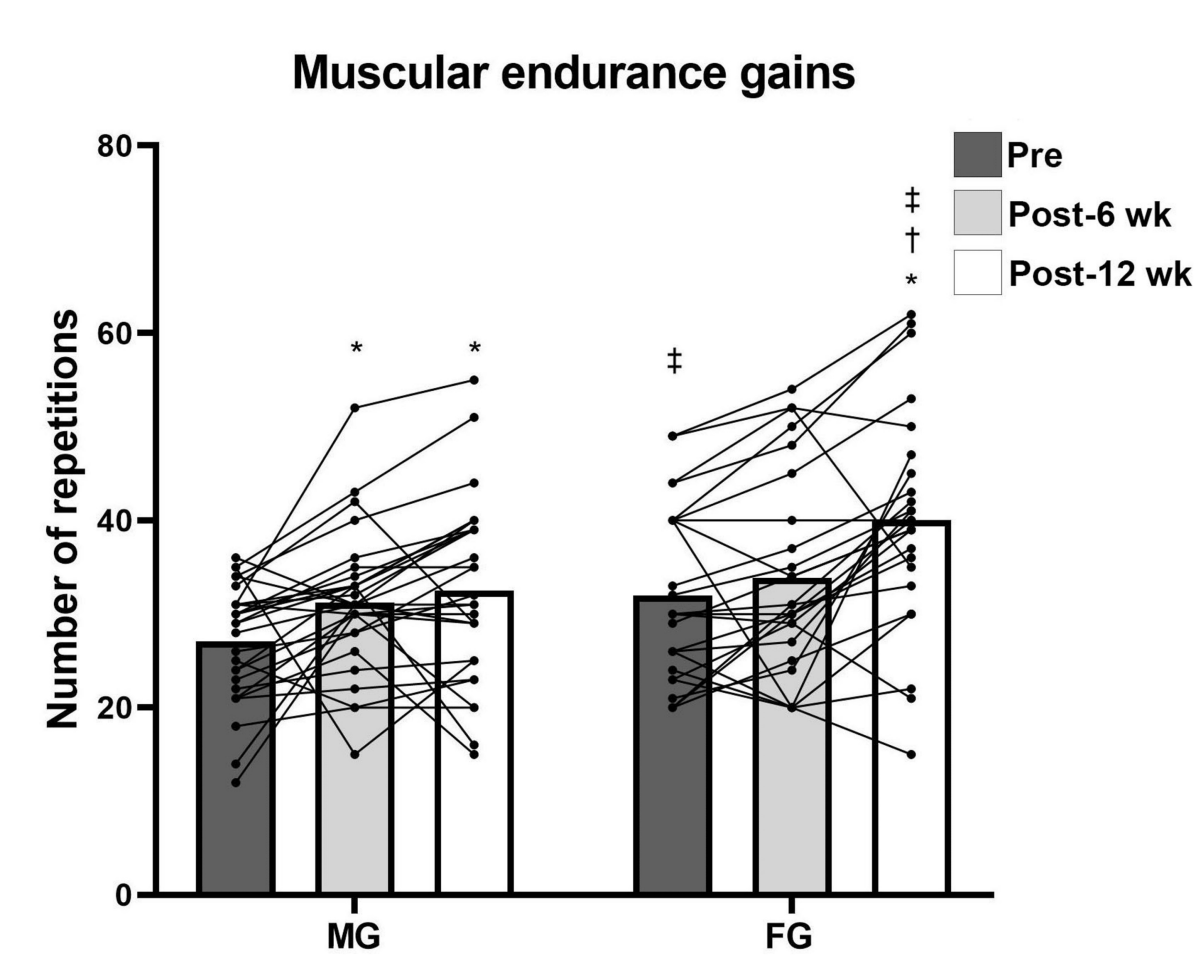
Deadlift muscular endurance pre- and post training for male group (MG) and female group (FG) *Significant difference from pre- to post training; †Significant difference from the sixth to 12th week (wk) of training; ‡ Significant difference between the sexes

**Figure 4:**
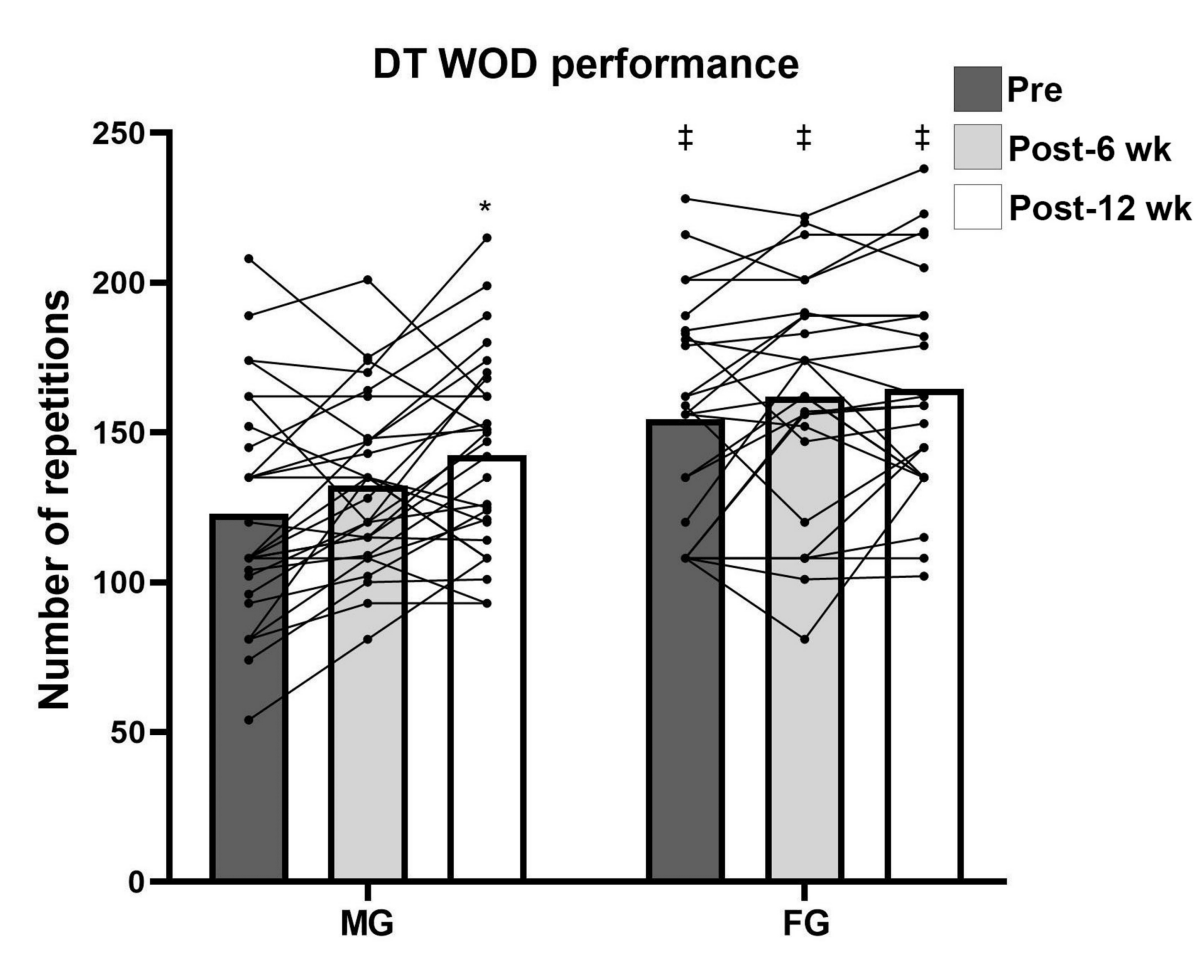
DT WOD pre- and post training for male group (MG) and female group (FG) *Significant difference from pre- to post training; ‡ Significant difference between the sexes WOD: workout of the day

Repeated measures analysis revealed significant pre- to post-test differences in both men and women for maximum strength (F (2, 102) = 94.534; p < 0.001) (Figure 1) and power (F (2, 102) = 41.821; p < 0.001), except in power for women from test 2 to test 3 (p = 1.0) (Figure 2). For muscular endurance, there were statistical pre- to post-test differences for both sexes (F (2, 102) = 18.920; p < 0.001), except for men from test 2 to test 3 (p = 1.0) and for women from test 1 to test 2 (p = 0.55) (Figure 3). For DT WOD, a statistical difference was revealed (F (2, 102) = 10.790; p < 0.001) from test 1 to test 3 only for men (p < 0.001) (Figure 4). Repeated measures analysis revealed statistical differences in maximum strength and jump height between men and women in all tests (p < 0.001). For muscular endurance and DT WOD, there were statistical differences between sexes, except for muscular endurance in test 2 (p = 0.29). Significant differences were identified between sex percentage change in the power test (t (51) = -2.240; p = 0.02) and in the DT WOD endurance test (t (51) = -2.018; p = 0.04). For maximum strength, the ES was large for men and moderate for women. For jump height, the ES was moderate for men and small for women. For muscular endurance, the ES was small for men and moderate for women. For DT WOD, the ES was small both for men and women (Table 2).

**Table 2:** Pre- and post training measures, effect sizes (ES), magnitudes, and percentage change pre- to post 12-week training MG: male group; FG: female group; Δ%: percentage change; 1RM: one repetition maximum; WOD: workout of the day *Significant Δ% difference between sexes

Variable	Groups	Pre-training	Post training	ES	ES magnitude	Δ% (95% CI)
Deadlift 1RM	MG	146.0 ± 22.3	163.6 ± 22.7	1.89	Large	12.6% (9.7 to 15.5)
	FG	92.4 ± 17.3	102.7 ± 18.7	1.14	Moderate	11.7% (7.3 to 16.0)
Jump height	MG	34.2 ± 5.1	38.4 ± 5.4	1.26	Moderate	12.9% (8.7 to 17.0)*
	FG	23.6 ± 4.6	25.1 ± 4.7	0.72	Small	6.8% (3.1 to 10.5)
Muscle endurance test	MG	27.0 ± 6.3	32.5 ± 9.7	0.61	Small	24.5% (8.2 to 40.8)
	FG	31.9 ± 9.3	40.0 ± 11.7	0.88	Moderate	29.2% (14.3 to 44.2)
DT WOD	MG	122.8 ± 37.5	147.4 ± 32.4	0.71	Small	21.3% (10.5 to 32.1)*
	FG	154.3 ± 38.1	164.4 ± 37.1	0.46	Small	1.3% (1.2 to 15.4)

## Discussion

The main objective of this study was to investigate whether there are sex differences in strength, power, and muscular endurance gains following a 12-week kettlebell swing protocol. Both sexes showed pre- to post-test differences for strength, power, and muscular endurance deadlift tests. In DT WOD, only men improved performance following 12-week kettlebell swing training. Furthermore, men had greater gains in the power rest and the DT WOD endurance test.

Previous studies have investigated strength and power adaptations using the same kettlebell protocol as the present study. Santos Junior et al. (2022) [20] divided 22 males into periodized (using a progressive intensity regime) and non-periodized groups (using the same intensity during the intervention period). After six weeks, both groups showed significant increases in deadlift 1RM (8.7% for the non-periodized group and 7.8% for the periodized group) and jump height (8.7% for the non-periodized group and 10.1% for the periodized group). Lake and Lauder (2012) [21] also investigated healthy men over a six-week period. Participants used fixed intensities in the kettlebell protocol and obtained significant increases in half squat 1RM (12%) and jump height (15%). In the present study, men increased 12.6% in the 1RM test and 12.9% in the jump height test. The duration of the present study may explain the greater results when compared to the study by Santos Junior et al. [20] (12 weeks versus six weeks). Despite the shorter intervention period in the study by Lake and Lauder [21], the results were similar for strength and superior for power when compared to the present study. These responses can be explained by the difference in training status (baseline values for jump height of 20 cm x 34.2 cm in the studies by Lake and Lauder [21] and Santos Junior et al. [20], respectively), since individuals with lower training levels may show superior responsiveness to training. It is not feasible to compare the responses between sexes after a kettlebell training protocol since, to our knowledge, no study has investigated this outcome.

Muscular strength and power increases in response to resistance training are influenced by both neural and muscular factors [6, 26]. To the best of our knowledge, no study has investigated the effect of kettlebell swing on hypertrophy, which makes it difficult to know the influence of this outcome on strength and power gains. Even when considering that there might be hypertrophic adaptations following a period of performing the kettlebell swing and that this could have contributed to strength and power gains, the literature shows that there seems to be no difference between sexes following a period of resistance training [6]. Whereas the contribution of the kettlebell exercise to increase deadlift performance lies in challenging time-limited force expression and differentially challenging motor demands [20], knowing the neural aspects between sexes is important to explain the results of the present study. On this issue, previous studies have shown that there were no significant differences between sexes in the motor unit activation [4], force per cross-sectional area (CSA), and contractile velocity of Type I and Type II fibers on a single fiber level [27]. Potential sex similarities in both muscular and neural aspects may explain the equivalent positive responses regarding strength gain following 12 weeks of kettlebell swing training. The results of the present study agreed with a systematic review and meta-analysis, which showed no differences between men and women for lower-body strength gains [6].

Similarities in both muscular and neural parameters do not explain significant sex differences for power gains across sexes, where men increased 12.9% (ES moderate) and women 6.8% (ES small). These results partially differ from the results of the article by Jozsi et al. (1999) [7], which compared power responses between sexes on the arm pull and leg extension machines at 40, 60, and 80% of 1RM before and following 12 weeks of resistance training. Both sexes increased arm pull power similarly, but men responded with greater absolute gains in leg extensor power at 40 and 60% of 1RM. Possible explanations for the differences in the results between the studies could be the training status, the type of training, and the tests used, since in the study by Jozsi et al. (1999) [7], subjects were sedentary and used traditional strength training exercises such as seated chest press, seated arm pull, unilateral knee extension, seated bilateral leg curl, and seated bilateral leg press.

In another study, conducted by Fernandez-Gonzalo and colleagues (2014) [8], 16 men and 16 women were assessed in jump height and power performance in the leg press at 50%, 60%, 70%, and 80% of 1RM. After six weeks of flywheel supine squat training, there was no difference between groups for jump height; however, the improvement in power performance at 80% of 1RM was greater for men, showing that even similar training stimuli can lead to subtle differences in power adaptations across sexes. Still, considering the absence of results for the other outcomes, the study by Fernandez-Gonzalo et al. [8] shows predominantly different results from the present study.

One of the potential explanations for the results found in power gains between sexes in the present study is the mass of the kettlebell used, as males used an average of 20 kg (approximately 25% of average body mass) and females used an average of 12 kg (approximately 18% of average body mass), and this may have generated a different training stimulus and influenced the sex-based adaptations. Indeed, it is possible that training prescriptions for women aiming at power gains should consist of a higher volume than currently used to induce adaptations in men [28]. Another possible explanation for the differences is the exercise used. Jones et al. (2016) [29] compared sex differences in muscular power in the deadlift exercise at 30%, 60%, and 90% of 1RM. The authors verified that men produced higher absolute average and peak power across all loads. The present study used the kettlebell swing as a training mode, but considering the angular displacement of the joints involved in the exercises, the swing and the deadlift show great similarities from a biomechanical perspective [20]. If the greater power production found in men in the deadlift also occurs in the kettlebell swing, this may explain the greater power gain found for men. Further work is needed in this area to better understand the mechanisms behind these responses.

Regarding the muscle endurance test, both men and women improved following 12 weeks of kettlebell training, with no significant difference between groups. However, for the DT WOD, which is also a muscular endurance test, only men improved following 12 weeks, and this difference was significant compared to women. As confirmed in the present study, women tend to resist fatigue more than men, and one of the explanations for this phenomenon is the greater glycolytic capacity in men and greater potential for oxidative metabolism in women [3]. One of the possible reasons for the improvement in the muscular endurance test is the increase in strength; however, both men and women increased strength, but only men improved DT WOD performance. It is important to emphasize that the DT WOD involves other movements, mainly for upper limbs, such as the hang power clean and push jerk, which involve muscles that are not primarily focused on the kettlebell swing, and therefore a training program aimed at improving the DT WOD should also involve other exercises. Considering the high cardiorespiratory demand resulting from the swing protocol [22], another possible explanation for the present result, specifically for the DT WOD, is that 12 weeks of kettlebell training was enough to generate an increase in the oxidative capacity and muscle mitochondrial enzyme machinery predominantly in the men. Furthermore, the fact that men trained with heavier kettlebells (both absolutely and relatively), generating a potentially greater training stimulus, may also explain the results.

The results of the present study regarding muscular endurance somewhat differ from other studies found in the literature, which either did not see a difference between sexes [9] or found greater gains in women [10,11]. However, all these studies were carried out with individuals who were sedentary (who did not practice any physical exercise for at least six months). Furthermore, to measure muscle endurance, these studies used a different protocol than the present study (four sets at 80% of 1RM to failure) and different exercises (Ribeiro et al. [9] used bench press and arm curl, and the other two studies used bench press, squat, and arm curl), and these factors may explain the divergent results.

There may be some possible limitations in this study, such as the fact that the nutritional status and the time of day that the subjects performed the tests were not controlled, which reduces the internal validity of the study. Likewise, the menstrual cycle was also not controlled. Future studies may investigate elderly individuals, use a larger sample, and especially focus on investigating biochemical and histological outcomes for a better understanding of the mechanisms responsible for the possible differences in adaptations between sexes for muscle power and muscular endurance on DT WOD.

## Conclusions

Kettlebell training appears to be an effective strategy for increasing muscle strength, power, and endurance in the deadlift test in both men and women. Considering that men obtained greater gains in power and in DT WOD, and only men improved in the DT WOD endurance test following 12 weeks, other strategies should also be considered to optimize women's results in these tests, such as using a greater training load (by increasing volume or intensity or both), using other modes and methods of training, and monitoring individual responses to understand which strategies are most suitable for each individual. Further research with biochemical and histological measures is required to clarify why these differences occurred and to seek strategies to enhance women's results on the aforementioned tests.
